# Can Behavioral Interventions Promote Health Equity in Pediatric Type 1 Diabetes? A Narrative Review of Promising Treatments

**DOI:** 10.1007/s11892-026-01629-2

**Published:** 2026-05-30

**Authors:** Paul T. Enlow, Jaquelin Flores Garcia, Julia Price, Kristen A. Torres, David V. Wagner

**Affiliations:** 1 Center for Healthcare Delivery Science, Nemours Children’s Hospital Delaware, Wilmington, DE USA; 2 Department of Pediatrics, Sidney Kimmel Medical College at Thomas Jefferson University, Philadelphia, PA USA; 3 Center for Endocrinology, Diabetes, & Metabolism, Children’s Hospital Los Angeles, Los Angeles, CA USA; 4 Department of Pediatrics, Oregon Health & Science University, Portland, OR USA

**Keywords:** Type 1 Diabetes, Pediatrics, Health Disparities, Behavior Treatment

## Abstract

**Purpose of Review:**

Synthesize data on whether existing behavioral interventions can improve health equity among youth with type 1 diabetes.

**Recent Findings:**

While existing behavioral interventions demonstrated efficacy in improving health and/or psychosocial outcomes, evidence that these same interventions may improve health equity were lacking. Most interventions were evaluated using predominantly White and affluent samples and some studies did not report on racial, ethnic, or sociodemographic characteristics of their sample. Only a few interventions have been adapted for youth from minoritized backgrounds. Recent multisystemic and technology/mHealth interventions recruited samples that were sociodemographically representative of youth experiencing disparities, suggesting that these interventions could improve health and/or psychosocial outcomes for these sociodemographic groups. However, no studies conducted subgroup analyses to examine whether the effect of the intervention might vary as a function of sociodemographic characteristics.

**Summary:**

The prevalence and persistence of disparities in psychosocial and glycemic outcomes among youth with T1D underscore the urgent need for effective, evidence-based behavioral interventions for populations in most need of this care. There is a critical need for research that prioritizes recruitment of samples that represent youth most impacted by health disparities. Additionally, existing interventions can be adapted for youth from minoritized backgrounds. Future research would benefit from leveraging existing intervention development/adaptation frameworks and engaging community partners through the research process.

## Introduction

Challenges with regularly completing the complex self-care regimen for type 1 diabetes (T1D) are common among youth and contribute to elevated glycemic levels and short- and long-term health complications [1–5]. Additionally, youth with T1D are at increased risk for adverse psychosocial outcomes compared to youth without T1D (e.g., depression, family conflict, quality of life) [6]. Behavioral interventions tailored to youth with T1D improve health outcomes by supporting youth and their caregivers in coping with the stresses of managing T1D, improving family interactions, and increasing self-care. These interventions vary in format and targets, ranging from interventions delivered to individual families to shift families patterns of communication (e.g., Behavioral Family Systems Therapy for Diabetes [7]) to multi-family interventions to promote problem-solving [8]; some interventions involve shared medical appointments [9], while others are multisystem interventions that address social determinants of health (SDOH) as well as self-management behaviors [10]. Despite their heterogeneity, there is strong evidence that behavioral interventions can improve the health and well-being of youth with T1D [11]. A growing body of literature has highlighted sociodemographic subgroups of youth with T1D that shoulder a disproportionate disease burden and would likely benefit most from behavioral interventions.

Sociodemographic disparities in pediatric T1D care and outcomes are profound, prevalent, and persistent. Compared to youth identifying as White or from more affluent families, youth who identify as Black or Latino/a or come from families of lower socioeconomic status are more likely to have an HbA1c above the 7% target recommended by the American Diabetes Association [12–14]. Furthermore, these populations have less access to diabetes technology such as insulin pumps and continuous glucose monitors (CGM) [13, 15], and are more likely to present to the emergency department or be hospitalized due to diabetic ketoacidosis (DKA) or other T1D-related complications [16]. Inequities in T1D care and outcomes are multiply determined with distal factors (e.g., social drivers of health [SDoH], health insurance coverage, systemic racism) influencing access to care and presenting as barriers to diabetes self-management [17]. For example, these factors may affect glycemic levels directly (e.g., health insurance not covering CGM), or indirectly through day-to-day challenges (e.g., financial insecurity requiring caregivers to work more than one job and limiting caregiver supervision). While upstream interventions that target more distal factors are needed to address the root causes of inequities in T1D care and outcomes, behavioral interventions may be able to mitigate inequities in T1D care and outcomes by addressing proximal (e.g., intensive skills training and reinforcement of diabetes care tasks, facilitating family collaboration around diabetes self-management) and distal determinants (e.g., identifying SDoH influence on diabetes management and problems-solving relevant resource access, partnering with medical teams to modify communication strategies and clinic policies that create barriers to family engagement and technology access). However, our understanding of whether behavioral interventions can promote health equity is limited.

Recent reviews have attempted to capture the strength of behavioral health interventions for youth with T1D, with some looking specifically at how interventions have been modified for and/or tested on youth from groups that are historically and currently discriminated against. Dimentstein and colleagues [18] conducted a systematic review of existing behavioral interventions for T1D with a focus on race/ethnicity factors. They found that, out of 24 studies, approximately two thirds reported on the racial and ethnic make-up of sample, over half recruited samples that were over 80% non-Hispanic White, and only 1 study specifically examined the impact on youth from marginalized racial and ethnic groups. Ellis and Naar [19] reviewed T1D interventions where over 50% of the enrolled youth were Black or Latino/a youth or families. They found that very few interventions are tailored to youth with minoritized backgrounds. Additionally, most papers reported on proof-of-concept and pilot studies with limited evidence of efficacy. Both reviews highlight the need for more specific recruitment and enrollment of youth from marginalized backgrounds in T1D behavioral intervention development and evaluation. Beyond race and ethnicity, evidence indicates that youth from lower socioeconomic status (SES) backgrounds, from rural communities, and with other marginalized identities experience health disparities [20–22]. However, we are not aware of any recent reviews that examine health inequities across marginalized populations. To fill this gap in the literature, the current narrative review aims to synthesize data supporting existing behavioral interventions to evaluate if these interventions also improve equity in health and psychosocial outcomes among pediatric T1D populations.

## Review Strategy

For the purposes of this review, we defined behavioral interventions as an intervention that aims to change some behavior related to T1D (e.g., blood glucose monitoring, coping with diabetes-related distress, communicating with a caregiver about diabetes). We included various intervention modalities (e.g., in-person, telehealth, mHealth) as well as interventions designed to influence uptake and sustainment of diabetes technology use. As this was a narrative review, we primarily relied on our own expertise in this area, discussions with colleagues with similar expertise, and examination of previously published systematic and scoping reviews. We also conducted a specific search of recent literature to ensure we captured studies published in the past 5 years that either we were not aware of and/or were not included in prior reviews. Papers reporting on clinical trial protocols, intervention development/adaptation, or intervention acceptability and feasibility (without reporting on post-treatment outcomes) were not reviewed.

Similar to Hilliard and colleagues’ review [11], we grouped behavioral interventions into four categories: skills training programs, family interventions, multisystemic interventions, and technology mHealth interventions (Table 1). Skills training programs were defined as interventions that either 1) directly used behavioral intervention s (e.g., reward systems, task building) to increase frequency of diabetes care tasks and/or sought to teach youth skills (e.g., coping, problem-solving) that can facilitate a variety of T1D self-management tasks (e.g., bolusing, nutrition). We considered interventions that target family interactions (e.g., communication, collaboration) as “family interventions.” Multisystemic interventions were required to target at least 2 or more systems in addition to the youth, parents, and families. Finally, technology mHealth interventions were defined as any intervention that was delivered electronically, including technology-mediated version of other interventions (e.g., family intervention delivered virtually). For each category of behavioral intervention, we examined: (1) existing interventions published in the literature, (2) the evidence supporting the efficacy or effectiveness of the intervention, (3) sociodemographic representation in intervention trials, and (4) indications of behavioral interventions improving health equity (e.g., subgroup analysis, sociodemographic predictors of response to treatment).

**Table 1:** Representative Interventions

Intervention	Author & year	Study design & *N*	Race/Ethnicity	SES variable(s)^a^
Skills Training Programs				
**PRISM**	Yi-Frazier et al., 2024^24^	RCT (*n* = 172)	White: 72.6%; Latino: 19.2%; Black: 15.1%; American Indian/Alaska Native/Asian: 6.3%; Multiracial/Other: 5.8%	*Insurance* Private: 75% Public: 25% Other: 1%
**FLEX**	Mayer-Davis et al., 2018^25^	RCT (*N* = 258)	White: 78%; Latino/a/x/e: 12.8%; Other: 5.4%; Black: 4.6%	*Insurance* Public: 18%
**STePS**	Weissberg-Benchell et al., 2020^26^	RCT (*N* = 264)	White: 65.5%; Black: 14.4%; Latino/a/x/e: 11.0%; Other: 5.7%; Asian/Pacific Islander: 2.3%; American Indian or Alaska Native: 1.1%	*Family Income* <$50k: 14.8% $50-$75k: 14.4% $76-$100k: 16.3% $101-150k: 18.9% >$150k: 23.9% *Maternal Education* College graduate: 61.4% *Home Structure* Two-parent home: 59.8%
**CST**	Grey et al., 2000^28^	RCT (*N* = 77)	White: 92% White; Black or Latino/a/x/e: 8%	*Household Income* <$29k: 17.3% $30k-74k: 60% >$75k: 16%
**BREATHE-T1D**	Basch et al., 2024^27^	RCT (*N* = 42)	White: 45.2%; Black: 26.2% Multicultural: 16.7%; Latino/a/x/e: 11.9%; Other: 7.1%; Asian: 2.4%; Native Hawaiian/Pacific Islander: 2.4%	*Household Income* < 25k: 2.4% $25-89k: 28.5% $90-$149k: 23.8% ≥$150k: 45.2% *Caregiver Education* HS or less: 9.5% Some college or trade: 21.4% College or grad school: 69%

Family Based Interventions				
**BFST-D**	Wysocki et al., 2007^7^	RCT (*N* = 104)	White: 53%; Black: 34%; Latino/a/x/e: 6%; Other: 6%	*Intact family* BFST-D: 43%; Standard Care: 41%; Edu. Support: 43% *Hollingshead SES Index* BFST-D: 40.4 (± 14.2); Standard Care: 40.1 (± 11.6); Edu. Support: 40.4 (± 13.7)
**Teamwork Intervention**	Anderson et al., 1999^33^	RCT (*N* = 85)	No reported race or ethnicity	*Two parent household*: 79%
	Laffel et al., 2003^32^	RCT (*N* = 105)	No reported race or ethnicity	*Two parent household* Intervention: 78%; Standard care: 92%
	Holmes et al., 2014^35^	RCT (*N* = 226)	*Percent of youth non-white* Control group: 24.7% Intervention group: 32.1%	*Non-intact family* Control: 22.7%; Intervention 23.7% *Hollingshead SES Index* Control: 48.7 (± 11.0); Intervention: 46.2 (± 11.22)
**Care Ambassador**	Svoren et al., 2003^34^	RCT (*N* = 299)	No reported race or ethnicity	No reported SES variables
	Katz et al., 2014^36^	RCT (*N* = 153)	Standard care: 98% White; Care Ambassador plus: 85% White; Care Ambassador plus ultra: 90% White	*Caregiver Education* Standard care: 69% had college degree or higher Care Ambassador plus: 67% college degree or higher Care Ambassador plus ultra: 64% college degree or higher

Multisystemic Interventions				
**MST**	Ellis et al., 2005^39^	RCT (*N* = 127)	*MST*: Black: 69%; White: 20%; Other: 11% *Control*: Black: 57%; White: 32%; Other: 11%	*Family Income* $28,437 (± $18,617) *Family Structure* Two-caregiver home: 57%
	Ellis et al., 2010^49^	RCT (*N* = 49)	Black: 100%	*Family Income* $29,598 *Family Structure* Two-caregiver home: 45% *Caregiver education*: College: 20%
**NICH**	Wong et al., 2025^44^	Prospective Observational (*N* = 26)	Black: 42%; Latino/a/e/x: 31%; Multiracial/Other: 15%; White: 8%; Native Hawaiian/Pacific Islander: 4%	*Insurance* Public: 96% *Social risk factors* ^*a*^ 8.0 ± 4.7 social risk factors at baseline
	Stover-Kempers et al., 2025^45^	Retrospective observational (*N* = 101)	*Intervention*: White: 85%; Latino/a/x/e: 12.5%; Multiracial: 7.5%; Black: 5%; Pacific Islander: 2.5% *Comparison*: White: 86.9%; Latino/a/x/e: 11.5; Multiracial 4.9%; Black: 1.6%; Pacific Islander: 1.6%; Asian: 1.6%	Over 95% of families and youth in the NICH program were living in poverty and experienced various related risks.

Technology and Mobile Health				
**Type 1 Doing Well App**	Hilliard et al., 2020^51^	RCT (*N* = 84)	Non-Latino/a/x/e white: 61%; Black: 13%; Other or multiracial: 7; Latino/a/x/e: 19%	*Insurance* Private insurance: 74%
**eHealth Intervention**	Ellis et al., 2024^52^	RCT (*N* = 149)	Black: 93.3%; Other: 6.7%	*Caregiver Education (average)*: 13.4 yrs *Family income (average)*: $34,933
**MyT1DHero**	Holtz et al., 2021^53^	Single-arm clinical trial	White: 88%; Black: 8%; Latino/a/x/e: 4%	*Insurance* Private: 72% *Family Income* ≥ $100,000: 40%
**Video-Based Telehealth Intervention**	Patton et al., 2021^54^	Non-randomized controlled pilot trial	Non-Latino white: 86.5%	None reported
**CoYoT1/Team Clinic**	Bisno et al., 2023^56^	RCT (*N* = 68)	*Race* White: 42%; Unknown: 19%; Multiracial: 12%; Native American: 12%; Asian: 6% *Ethnicity* Latino/a/e/x: 50%	*Insurance* Public: 75%
	Flores Garcia et al., 2024^59^	Pragmatic randomized controlled trial (*N* = 79)	White: 36%; Latino/a/e/x: 34%; Other/Unknown: 10%; Asian: 8%; Black: 6%; Multiracial: 5%; Hawaiian/Pacific Islander: 1%	*Public Insurance*: 20% *English primary language*: 92% *Caregiver education*: 42% college or greater *Caregiver marital status*: 56% married/civil union
	Flores Garcia et al., 2025^55^	RCT (*N* = 68)	*Race* White: 42%; Unknown: 19%; Multiracial: 12%; Native American: 12%; Black: 9%; Asian: 6% *Ethnicity* Latino/a/e/x: 50%	*Insurance* Public: 75%
**REAL-T**	Pyatak et al., 2025^57^	Two-arm parallel-group randomized controlled trial (*N*= 209)	White: 44.5%; Latino/a/e/x: 43.5%; Black: 3.8%; Multiracial: 3.8%; Asian: 1.9%; Other: 1.9%; Decline to answer: 0.5%	*Public insurance*: 47.4% *Employed full time*: 43.1% *Social Deprivation Index*: 63.52 (± 27.46) *Social Vulnerability Index*: 0.56 (± 0.29) *Social needs*: 2.10 (± 1.83)
**3Ms**	Ellis et al., 2017^58^	RCT (*N* = 67)	Black: 100%	*Median Family income*: $20k-$29k (IQR: $10k-$19k, $40k-$49k)

*Notes*: ^a^For ease of reading, some categories have been combined or omitted if options were binary (e.g., public vs. private insurance); ^b^Social Risk factors include educational barriers, mental health needs, trauma, food insecurity, etc.;

## Results

### Skills Training Interventions

Utilizing multifaceted approaches, recent skills training interventions have focused on problem-solving skills, resilience and coping, and motivation [23–27]. For example, Yi-Frazier and colleagues [24] tested the efficacy of Promoting Resilience in Stress Management (PRISM), a primarily youth-focused intervention utilizing short skill-based modules focused on stress management and mindfulness, goal setting, cognitive restructuring, and benefit finding. Mayer-Davis and colleagues developed and tested the Flexible Lifestyles Empowering Change (FLEX) Intervention which used motivational interviewing and problem-solving skills to enhance diabetes self-management [25]. Weissberg-Benchell and colleagues adapted the Penn Resilience Program, an intervention focusing on building hope and optimism, for youth with T1D (PRP T1D) [26]. An additional intervention, Coping Skills Training (CST), taught youth how to cope with stressful life situations in relation to managing T1D care [28]. Lastly, Basch and colleagues developed and tested BREATHE-T1D, a youth focused mindfulness-based group intervention aimed at reducing stress that was adapted for youth with T1D [27].

#### Efficacy and Effectiveness

Skills training interventions demonstrated statistically significant improvements in patient reported outcomes with less robust findings related to HbA1c. PRISM reduced youth diabetes distress and improved self-management behaviors [24]. Participants in the original and internet-based CST reported improvements in quality of life and coping skills. Mayer-Davis and colleagues found that FLEX improved motivation, problem-solving, diabetes self-management, and quality of life [25]. PRP T1D reduced diabetes distress and depressive symptoms [26]. Lastly, BREATHE-T1D resulted in statistically significant reductions in depressive symptoms [27]. Although not always the primary goal of these interventions, some skills training interventions improved glycemic levels. BREATHE-T1D [27] and CST demonstrated a reduction in HbA1c [28], although CST no longer showed improvements in HbA1c once adapted for internet use [29]. PRISM, PRP T1D, and FLEX were not associated with statistically significant reductions in HbA1c from baseline to post-treatment timepoints [24–26].

#### Sociodemographic Representativeness in Trials

Across most studies, the racial and ethnic makeup of the sample was generally representative of the broader clinical population of youth with T1D [30]. In the clinical trials of PRISM, FLEX, the majority of adolescents (65–77%) identified as non-Latino/a, White [24–26]. The 2000 CST was majority White (92%) with the other reported race/ethnicity being combined African American/Black and Latino/a/x/e (8%). In the 2013 version of CST, the majority of the sample was White (62.2%) with the other reported race/ethnicity being combined African American/Black and Latino/a/x/e (37.8%). The sample for BREATHE-T1D was more racially diverse with the majority of the sample identifying as Black/African American, Biracial, or another minoritized racial groups (i.e., Asian, Pacific Islander, or another race); most participants (> 80%) identified as non-Latino [27]. Variables used to characterize the socioeconomic status of participants and their families ranged from public insurance to family income to caregiver education. Across interventions, the samples were predominantly socioeconomically advantaged (e.g., private insurance, affluent, post-secondary education) [23–27].

#### Indications of Impact on Equity

While the samples included in skills training program evaluations were generally representative of the broader T1D population, there was no evidence with any of the trials that their respective interventions improved health equity.

### Family-Based Interventions

Family factors such as caregiver involvement in monitoring of youth diabetes self-management, caregiver support of adolescent autonomy in diabetes self-management, and family conflict have strong and enduring associations with glycemic levels [6]. While interventions are varied, family processes (e.g., communication, problem-solving) and behavior management strategies (e.g., goal setting, rewards and punishments) are commonly employed. Behavioral Family Systems Therapy for Diabetes (BFST-D) [7, 31] and Family Teamwork [32] are two of the most commonly studied family-based interventions.

#### Efficacy and Effectiveness

In the initial studies, BFST-D was found to lower glycemic levels and reduce family conflict but only for youth with an HbA1c > 9.0% at baseline [7, 31]. However, BFST-D did improve self-reports of adherence regardless of pre-treatment glycemic levels [7, 31]. Family Teamwork was shown to decrease family conflict and unsupportive family behaviors, lower glycemic levels, and reduce the rates of emergency department (ED) visits and hospitalizations [32].

#### Sociodemographic Representativeness in Trials

In the initial studies for BFST-D, Wysocki and colleagues reported on the racial/ethnic and socioeconomic characteristics of their samples [7, 31], which were racially and ethnically representative of the larger population of youth with T1D [7, 30, 31]. Studies reporting on the Family Teamwork intervention varied with regard to racial and ethnic representation. Earlier studies did not report on the racial and ethnic characteristics of their samples [32–34]. Later studies dichotomized race and ethnicity as white or non-white and were majority white (i.e., ≥ 75%) [35–37]. Descriptions of socioeconomic characteristics varied for BFST-D and Family Teamwork (e.g., Hollingshead Index, highest caregiver education, occupational status code). Regardless of the variable used, samples for both BFST-D and Family Teamwork trended towards more affluent families (e.g., Hollingshead Index ≥ 40, > 50% with college education or greater) [7, 31–37].

#### Indications of Impact on Equity

Neither BFST-D nor Family Teamwork studies performed subgroup analyses to determine whether the effect of these interventions varied as a function of patient race/ethnicity or socioeconomic status. Butler and colleagues adapted the Family Teamwork intervention for African American/Black and Latino/a caregivers of youth with T1D [38]. While the adapted intervention was highly acceptable, many caregivers had difficulty attending intervention sessions, a phenomenon that has been documented in other clinical trials of family-based interventions [37]. Due to the pilot nature of this study [38], evidence regarding the efficacy of the adapted intervention is not yet available.

### Multisystemic Interventions

Compared to interventions that target single (e.g., individual youth) or dual systems (e.g., caregivers, youth), multisystem interventions focus on areas both within and beyond the family system. Multisystemic Therapy adapted for youth with T1D (MST-T1D) and Novel Interventions in Children’s Healthcare [NICH] are both intensive home- and community-based interventions that were designed to target youth and families with high social needs. MST-T1D involves 48 home visits with adolescents and caregivers that aim to support challenges at the level of the adolescent, family system, and broader community (e.g., school, healthcare system) that interfere with diabetes self-care tasks and management [39]. NICH includes 24/7 access to an interventionist and targets SDoH-related barriers to specialty care attendance, diabetes technology access, and T1D care tasks [10]. NICH interventionists also attend diabetes appointments, partner with medical teams to tailor communication and recommendations to families’ lived experiences, and use technology to engage youth daily in evidence-based skills building interventions.

#### Efficacy and Effectiveness

Youth who were in the “high dose” MST group showed significantly greater improvements in HbA1c, fewer hospital admissions, and more frequent blood glucose checks compared to those receiving standard care [39]. In a 2-year follow-up study, investigators found that youth in MST engaged in more frequent blood glucose testing, had fewer DKA events and lower costs related to DKA compared to youth in usual services [40]. While HbA1c decreases were not significant when comparing MST youth to those in control group, MST youth from 2-compared to single-caregiver families demonstrated reduction in HbA1c over 2 years [41].

Although NICH has yet to be evaluated in a randomized controlled trial (RCT), multiple studies have examined program outcomes using quasi experimental designs. When examining within treatment group, NICH has been associated with improvements in youth health (i.e., clinically and statistically significant improvements in HbA1c), reductions in diabetes-related complications (e.g., DKA episodes) and associated acute utilization (i.e., ED visits, hospital admissions) [42, 43]. NICH is also associated with significant reductions in youth depressive symptoms and caregiver diabetes distress, as well as increases in diabetes-related strengths [44]. When examining NICH outcomes compared to similarly referred youth, electronic health record (EHR) data show that youth in NICH experienced a greater likelihood of reducing HbA1c below 10% and demonstrated a larger decrease in hospital admissions over time compared to youth who were referred to NICH but did not receive the program [45]. Furthermore, when evaluated using claims data, NICH demonstrated significant reductions in ED visits, admissions, and healthcare costs in the 3 years following enrollment compared to matched controls [46]. Finally, NICH has also demonstrated an ability to be implemented in healthcare systems beyond where initially developed, including in more racially and ethnically diverse communities [44, 47], with fidelity to the model and similar health outcomes.

#### Sociodemographic Representativeness in Trials

Over 60% of youth enrolled in MST were African American and over 60% were on public assistance. Similarly, NICH was specifically developed for youth experiencing inequities due to high social risk, with over 90% of youth on Medicaid and sample demographics being more racially and ethnically diverse than typical in region [48] (e.g., over 90% of youth in NICH in Bay Area from racial and ethnic groups that are historically marginalized [44]).

#### Indications of Impact on Equity

Ellis and colleagues have demonstrated intentionality in adapting the model specifically to meet the needs of under-resourced communities, and have shown in parallel work (i.e., MST for African American adolescents with high BMI) that they have used cultural adaptations identified by the community [49]. Similarly, NICH was intentionally developed to meet the needs of youth from historically minoritized backgrounds and who are disproportionately affected by health disparities [47] with evidence that delivering services to families in Spanish via bilingual providers continues to be associated with comparable improvements in health [50]. Taken together, both highlighted multisystem interventions have demonstrated the potential for improving the health and well-being of youth with T1D most impacted by health disparities and their families.

### Technology and Mobile Health (m-Health)

Technology and mHealth behavioral interventions can be delivered via various virtual modalities (e.g., videos, interactional software, telehealth) with a focus on youth and/or youth-family dyads [11, 51, 52].

*Application-based interventions.* In *Type 1 Doing Well*, caregivers add daily positive T1D-related actions into the app, which then generates a summary of strengths. Caregivers also have access to videos to learn how to recognize strengths and how to effectively provide praise to their youth [51].The *3M’s* uses an internet-based, interactional software and employs psychoeducational videos about 3 strategies to increase caregiver involvement (e.g., ensuring the caregiver watches the youth take insulin, monitors a blood glucose check, and eats 1 meal with their youth to assess carb counting) [52]. *MyT1D Hero* uses two interfaces, one for the caregiver and another for the youth, which aim to facilitate positive diabetes-specific communication about blood glucose schedules [53].

*Video-based telehealth interventions*. The *CARES* (Cognitive Adaptation to Reduce Emotional Stress) intervention aims to reduce caregiver diabetes distress through courses rooted in cognitive behavioral therapy, acceptance and commitment therapy, behavioral activation, and mindfulness [54]. *Team Clinic* and *The Colorado Youth with Type 1 Diabetes* (*CoYoT1)* (adapted for marginalized youth) also target psychosocial and diabetes care outcomes (e.g., diabetes distress, burnout) via motivational interviewing techniques, providing person-centered care, and access to virtual peer groups [55, 56]. The Resilient, Empowered, Active Living-Telehealth (REAL-T), a telehealth occupational therapy intervention for youth with T1D intervened across five content areas (habits & routines, managing T1D, emotions & well-being, understanding how contextual factors impact T1D management, and accessing healthcare) to improve psychological well-being, diabetes management, and health outcomes [57].

#### Efficacy and Effectiveness

Both *3M’s* and MyT1D Hero showed significant improvements in HbA1c, although only among those with higher initial depressive symptoms or greater use of the app, respectively. *CARES*,* Team Clinic*, and *REAL-T* trial results indicate improvements in depressive symptoms (*Team Clinic*,* CARES*), diabetes distress (*CARES*,* REAL-T*), resilience (*Team Clinic*) and quality of life (*REAL-T*). While caregivers expressed high satisfaction with *Type 1 Doing Well*, trial results indicated no statistically significant improvement in HbA1c [51].

#### Sociodemographic Representation in Trials

The samples for *Type 1 Doing Well*,* MyT1DHero*, and *CARES* were predominantly White families with private insurance and/or higher income [51, 53, 54]. To fill this gap some studies focused on marginalized communities. For example, two RCTs of the *3Ms* intervention included a cohort of approximately 93% and 89% Black youth, respectively, and with lower income [52, 58]. Recent studies of *CoYoT1*,* Team Clinic*, and *REAL-T* recruited samples with at least half of participants identifying as Latinx, and included participants from other sociodemographic and clinical groups experiencing historical inequities (e.g., Pacific Islander, Black, American Indian, Alaska Native, on public insurance) in comparison to the total non-white participants with private insurance [55–57, 59].

#### Indications of Impact on Equity

With the increased inclusion of racially-and-ethnically minoritized and low-income populations [55–57, 59] there is growing evidence that technology and mHealth interventions can improve psychosocial and health outcomes for these sociodemographic subgroups. Given the rapid growth of technology and mHealth interventions, further investigation will not only be needed to demonstrate benefits for marginalized communities but also the expansion in inclusion of multiple minoritized subgroup (e.g., LGBTA+, Indigenous).

## Discussion

The current narrative review examined recent literature to discern the potential for behavioral interventions to improve equity in health and psychosocial outcomes among youth with T1D. Unfortunately and unsurprisingly, youth from minoritized sociodemographic groups remain under-represented in most clinical trials. Studies that were conducted more recently and those that evaluated multisystem interventions tended to enroll more racially, ethnically, and socioeconomically diverse samples. Importantly, no studies performed subgroup analyses or examined whether interventions reduce inequities in health outcomes.

The prevalence and persistence of disparities in psychosocial and glycemic outcomes among youth with T1D underscore the urgent need for effective, evidence-based behavioral interventions for populations in most need of this care. Development of novel interventions specifically designed for underrepresented populations (e.g., racially and ethnically minoritized youth, LGBTQ + and non-binary youth) is one key pathway to address this gap [19, 60]. Indeed, adapted versions are already being developed for historically underrepresented populations (e.g., Butler and colleagues’ adaptation of Family Teamwork [38]). Interestingly, while more recent clinical research studies have demonstrated success in enrolling youth from racial and ethnic groups that have been historically underrepresented, we did not find similar trends for LGBTQ+ groups and struggled to identify many trials that reported gender beyond the binary. We also noted that even those interventions explicitly trying to engage youth from minoritized groups rarely reported use of community-based participatory research (CBPR) methods to inform intervention and study development. Engaging youth and families from minoritized groups in intervention development, evaluation, and implementation can ensure that interventions are tailored to community needs, more acceptable, and engender trust in the families receiving the intervention [60–62]. Future research in this area may involve applications of the Transcreation Framework [63], an implementation science framework for community-engaged behavioral interventions to reduce health disparities. There are also examples of how to adapt interventions for [64, 65], and engage with [66], marginalized communities that researchers can employ in their projects [67].

Another way to address this gap in the literature is to ensure that trials of behavioral interventions include substantial proportions of youth with T1D from various demographic backgrounds [68], particularly those who are underrepresented in research and often experience disparities. Studies evaluating the effectiveness of an intervention should aim to recruit samples that are reflective of the target population (e.g., those with higher HbA1cs, greater diabetes distress) rather than the general T1D population. Researchers should employ multiple strategies to recruit more representative samples [69], such as: community engagement in the form of working with parental advisory boards for feedback on recruitment materials [70], culturally relevant recruitment materials [71], culturally and linguistically congruent research team members [72], and flexible recruitment hours [69]. Researchers would benefit from these practices to further capture how interventions yield results in vulnerable populations. While promising evidence-based interventions exist, data are lacking on how these interventions perform among racially and ethnically diverse families and families from lower income of any race or ethnicity. It remains unclear if and how these interventions may be altered to optimize outcomes for underrepresented populations. Engaging more representative samples in intervention development and evaluation can address these important scientific gaps and make the interventions more applicable to the youth and families who are most likely to receive them as part of clinical care.

While developing new or adapting existing interventions to improve health equity requires intentionality from treatment developers and researchers, such efforts ultimately rely upon funding bodies to both prioritize and adequately fund such studies. While we highlight the lack of examining differential outcomes for different subgroups, such an approach ultimately relies on large enough sample sizes, time to intentionally engage communities and modify materials, and the funding support necessary to engage in such efforts. Historical and current funding levels for behavioral health research in T1D pale in comparison to medical funding and are often inadequate for health equity intervention research – such that treatment developers often first ask “will this treatment work for those I can easily enroll” as opposed to “will this work across groups and particularly for those who most need it.” Anecdotally, we have experienced submitting proposals to fund interventions targeting diverse populations only to be told that such proposals should be scaled back to meet funder balance sheets.

Multisystem interventions (NICH, MST) have demonstrated the most evidence in improving outcomes among youth and families experiencing these disparities. MST-T1D has been tested in multiple clinical trials with marginalized populations and long-term outcomes, and NICH has shown promise in implementing in new regions with racially, ethnically, and socioeconomically diverse populations. In line with intervention development frameworks, there is a need for efforts to disseminate and implement multisystem interventions more broadly. Identifying nontraditional reimbursement methods, such as partnering with insurance payors [48] or embedding programs at no cost to families in children’s hospitals as standard of care may provide financial support for offering programs more broadly [48] as these intervention models require more flexibility and intensity than current payment structures allow for. Further, local, state, and national government would benefit from investing in programs such as these during childhood as opposed to waiting to instead pay later for avoidable problems (e.g., emergency department visits for DKA, chronic medical complications, unemployment). Additionally, future research could examine the possibilities for establishing collaborative partnerships with state mental health services and more robust community mental health programs that already offer multisystem interventions.

While our narrative review has multiple strengths (e.g., examining evidence for equity beyond race and ethnicity, focusing on promising behavioral interventions, inclusion of health and psychosocial outcomes), our findings should be taken in the context of certain limitations. First, the decision to conduct a narrative review instead of a systematic review presents methodological limitations and may have resulted in the unintentional omission of relevant studies. Second, interventions that focused on practice change and behavioral economics (i.e., paying patients for completing self-management tasks) were not included but may be viable intervention strategies. Lastly, biases and limitations present within the scientific literature more broadly (e.g., limited publication of non-significant findings, unpublished studies, variability in instruments used to assess patient reported outcomes) will be present in our findings and conclusions as well. Also, of note, the vast majority of intervention studies included did explicitly aim to design interventions and/or research that addresses health equity, which may seem an unfair bar to overcome in absence of adequate funding.

## Conclusions

While more recent studies have been recruiting more sociodemographically diverse samples, evidence that behavioral interventions promote health equity in pediatric type 1 diabetes is limited. Multisystem and technology/mHealth interventions possessed the most evidence for improving outcomes among youth and families experience disparities. Analyzing treatment effects within sociodemographic subgroups or conducting studies that focus specifically on historically underserved populations has the potential to improve our understanding of how behavioral interventions may promote health equity. Additionally, there is a need for funders to support improved sociodemographic representation in intervention development and evaluation. 

## Key References

- Ellis DA, Naar S. Interventions Across the Translational Research Spectrum: Addressing Disparities Among Racial and Ethnic Minoritized Youth with Type 1 Diabetes. Endocrinol Metab Clin North Am. 2023;52(4):585-602.
  - ○ This systematic review identifies gaps in the literature regarding equity-promoting interventions and proposes novel models of developing interventions to reduce health disparities.
- Flores Garcia J, Reid MW, Pyatak EA, Fox DS, Bisno DI, Salcedo-Rodriguez E, et al. Person-Centered Care and Telehealth Reduce Distress and A1C for Marginalized Adolescents and Young Adults With Type 1 Diabetes. Clin Diabetes. 2025;43(2):270-81
  - ○ Highlights CoYoT1 as an intervention that can improve health outcomes among Latinx youth with T1D.
- Wong JC, Reed A, Noya C, Stone A, Spiro K, McGrath M, et al. Underresourced Youth With Diabetes in a Community-Based Intervention Show Improved Diabetes Outcomes, Technology Use, and Psychosocial Functioning. Endocr Pract. 2025;31(5):571-7.
  - ○ Demonstrates how full-scale implementation of NICH can improve health outcomes among underresourced youth.
- Wong JJ, Alamarie SA, Hanes SJ, Flores H, Ngo J, Schneider-Utaka AK, et al. An RCT of a family intervention for adolescents living with type 1 diabetes: Who benefits most? Diabet Med. 2025:e70151.
  - ○ Provides evidence that a family-based intervention had greater benefits for underresourced or racially and ethnically minoritized youth.

## Data Availability

No datasets were generated or analysed during the current study.

## References

[1] Peyrot M, Rubin RR, Kruger DF, Travis LB. Correlates of insulin injection omission. Diabetes Care. 2010;33(2):240–5. 20103556 10.2337/dc09-1348 PMC2809256

[2] Borus JS, Laffel L. Adherence challenges in the management of type 1 diabetes in adolescents: prevention and intervention. Curr Opin Pediatr. 2010;22:405–11. 20489639 10.1097/MOP.0b013e32833a46a7 PMC3159529

[3] Hood KK, Peterson CM, Rohan JM, Drotar D. Association between adherence and glycemic control in pediatric type 1 diabetes: a meta-analysis. Pediatrics. 2009;124(6):e1171–9. 19884476 10.1542/peds.2009-0207

[4] Gonzalez JS, Tanenbaum ML, Commissariat PV. Psychosocial factors in medication adherence and diabetes self-management: Implications for research and practice. Am Psychol. 2016;71(7):539. 27690483 10.1037/a0040388 PMC5792162

[5] Hunter CM. Understanding diabetes and the role of psychology in its prevention and treatment. Am Psychol. 2016;71(7):515. 27690481 10.1037/a0040344

[6] de Wit M, Gajewska KA, Goethals ER, McDarby V, Zhao X, Hapunda G, et al. ISPAD Clinical practice consensus guidelines 2022: Psychological care of children, adolescents and young adults with diabetes. Pediatr Diabetes. 2022;23(8):1373–89. 36464988 10.1111/pedi.13428 PMC10107478

[7] Wysocki T, Harris MA, Buckloh LM, Mertlich D, Lochrie AS, Taylor A, et al. Effects of behavioral family systems therapy for diabetes on adolescents’ family relationships, treatment adherence, and metabolic control. J Pediatr Psychol. 2006;31:928–38. 16401678 10.1093/jpepsy/jsj098

[8] Carpenter JL, Price JEW, Cohen MJ, Shoe KM, Pendley JS. Multifamily Group Problem-solving intervention for adherence challenges in pediatric insulin-dependent diabetes. Clin Pract Pediatr Psychol. 2014;2(2):101–15.

[9] Bisno DI, Reid MW, Fogel JL, Pyatak EA, Majidi S, Raymond JK. Virtual group appointments reduce distress and improve care management in young adults with type 1 diabetes. J Diabetes Sci Technol. 2022;16(6):1419–27. 34328029 10.1177/19322968211035768 PMC9631532

[10] Harris MA, Spiro K, Heywood M, Wagner DV, Hoehn D, Hatten A, et al. Novel Interventions in Children’s Health Care (NICH): Innovative treatment for youth with complex medical conditions. Clin Pract Pediatr Psychol. 2013;1(2):137–45.

[11] Hilliard ME, Powell PW, Anderson BJ. Evidence-based behavioral interventions to promote diabetes management in children, adolescents, and families. Am Psychol. 2016;71(7):590–601. 27690487 10.1037/a0040359 PMC5724567

[12] Addala A, Auzanneau M, Miller K, Maier W, Foster N, Kapellen T, et al. A decade of disparities in diabetes technology use and HbA1c in pediatric type 1 diabetes: a transatlantic comparison. Diabetes Care. 2021;44(1):133–40. 32938745 10.2337/dc20-0257 PMC8162452

[13] Ebekozien O, Mungmode A, Sanchez J, Rompicherla S, Demeterco-Berggren C, Weinstock RS, et al. Longitudinal trends in glycemic outcomes and technology use for over 48,000 people with type 1 diabetes (2016–2022) from the T1D exchange quality improvement collaborative. Diabetes Technol Ther. 2023;25(11):765–73. 37768677 10.1089/dia.2023.0320

[14] Demeterco-Berggren C, Ebekozien O, Noor N, Rompicherla S, Majidi S, Jones NY, et al. Factors associated with achieving target A1C in children and adolescents with type 1 diabetes: Findings from the T1D exchange quality improvement collaborative. Clin Diabetes. 2022;41(1):68–75. 36714245 10.2337/cd22-0073 PMC9845079

[15] Lipman TH, Smith JA, Patil O, Willi SM, Hawkes CPJPD. Racial disparities in treatment and outcomes of children with type 1 diabetes. 2021;22(2):241–8. 10.1111/pedi.13139 33871154

[16] Willi SM, Miller KM, DiMeglio LA, Klingensmith GJ, Simmons JH, Tamborlane WV, et al. Racial-ethnic disparities in management and outcomes among children with type 1 diabetes. Pediatrics. 2015;135:424–34. 25687140 10.1542/peds.2014-1774 PMC4533245

[17] Lipman TH, Hawkes CP. Racial and socioeconomic disparities in pediatric type 1 diabetes: Time for a paradigm shift in approach. Diabetes Care. 2021;44(1):14–6. 33444165 10.2337/dci20-0048

[18] Dimentstein K, Greenberg BA, Valenzuela JM. Involvement of racially and ethnically minoritized youths in behavioral type 1 diabetes interventions: A systematic review. J Pediatr Psychol. 2023;48(5):428–47. 37016949 10.1093/jpepsy/jsad018

[19] Ellis DA, Naar S. Interventions across the translational research spectrum: Addressing disparities among racial and ethnic minoritized youth with type 1 diabetes. Endocrinol Metab Clin North Am. 2023;52(4):585–602. 37865475 10.1016/j.ecl.2023.05.002

[20] Tilden DR, French B, Datye KA, Jaser SS. Disparities in continuous glucose monitor use between children with type 1 diabetes living in urban and rural areas. Diabetes Care. 2024;47(3):346–52. 37906202 10.2337/dc23-1564 PMC10909681

[21] Tatti P, Pavandeep S. Gender Difference in Type 1 Diabetes: An Underevaluated Dimension of the Disease. Diabetol [Internet]. 2022;3(2):364. 8 pp.].

[22] Gill A, Gothard MD, Briggs Early K. Glycemic outcomes among rural patients in the type 1 diabetes T1D Exchange registry, January 2016-March 2018: a cross-sectional cohort study. BMJ Open Diabetes Res Care. 2022;10(1):e002564. 35042753 10.1136/bmjdrc-2021-002564 PMC8768930

[23] O’Donnell HK, Vigers T, Johnson SB, Pyle L, Wright N, Deeb LC, et al. Pump It Up! A randomized clinical trial to optimize insulin pump self-management behaviors in adolescents with type 1 diabetes. Contemp Clin Trials. 2021;102:106279. 33440262 10.1016/j.cct.2021.106279 PMC8341128

[24] Yi-Frazier JP, Hilliard ME, O’Donnell MB, Zhou C, Ellisor BM, Garcia Perez S, et al. Promoting resilience in stress management for adolescents with type 1 diabetes: A randomized clinical trial. JAMA Netw Open. 2024;7(8):e2428287. 39158914 10.1001/jamanetworkopen.2024.28287 PMC11333977

[25] Mayer-Davis EJ, Maahs DM, Seid M, Crandell J, Bishop FK, Driscoll KA, et al. Efficacy of the flexible lifestyles empowering change intervention on metabolic and psychosocial outcomes in adolescents with type 1 diabetes (FLEX): a randomised controlled trial. Lancet Child Adolesc Health. 2018;2(9):635–46. 30119757 10.1016/S2352-4642(18)30208-6 PMC6260973

[26] Weissberg-Benchell J, Shapiro JB, Bryant FB, Hood KK. Supporting Teen Problem-Solving (STEPS) 3 year outcomes: Preventing diabetes-specific emotional distress and depressive symptoms in adolescents with type 1 diabetes. J Consult Clin Psychol. 2020;88(11):1019–31. 33136423 10.1037/ccp0000608 PMC8279264

[27] Basch M, Lupini F, Ho S, Dagnachew M, Gutierrez-Colina AM, Patterson Kelly K, et al. Mindfulness-based group intervention for adolescents with type 1 diabetes: initial findings from a pilot and feasibility randomized controlled trial. J Pediatr Psychol. 2024;49(10):769–79. 39212647 10.1093/jpepsy/jsae071 PMC11493139

[28] Grey M, Boland EA, Davidson M, Li J, Tamborlane WV. Coping skills training for youth with diabetes mellitus has long-lasting effects on metabolic control and quality of life. J Pediatr. 2000;137:107–13. 10891831 10.1067/mpd.2000.106568

[29] Grey M, Whittemore R, Jeon S, Murphy K, Faulkner MS, Delamater A. Internet psycho-education programs improve outcomes in youth with type 1 diabetes. Diabetes Care. 2013;36(9):2475–82. 23579179 10.2337/dc12-2199 PMC3747907

[30] Fang M, Wang D, Selvin E. Prevalence of Type 1 Diabetes Among US Children and Adults by Age, Sex, Race, and Ethnicity. JAMA. 2024;331(16):1411–3. 38573653 10.1001/jama.2024.2103 PMC11040401

[31] Wysocki T, Harris MA, Buckloh LM, Mertlich D, Lochrie AS, Mauras N, et al. Randomized trial of behavioral family systems therapy for diabetes: maintenance of effects on diabetes outcomes in adolescents. Diabetes Care. 2007;30(3):555–60. 17327320 10.2337/dc06-1613

[32] Laffel LM, Vangsness L, Connell A, Goebel-Fabbri A, Butler D, Anderson BJ. Impact of ambulatory, family-focused teamwork intervention on glycemic control in youth with type 1 diabetes. J Pediatr. 2003;142(4):409–16. 12712059 10.1067/mpd.2003.138

[33] Anderson BJ, Brackett J, Ho J, Laffel LM. An office-based intervention to maintain parent-adolescent teamwork in diabetes management. Impact on parent involvement, family conflict, and subsequent glycemic control. Diabetes Care. 1999;22(5):713–21. 10332671 10.2337/diacare.22.5.713

[34] Svoren BM, Butler D, Levine BS, Anderson BJ, Laffel LM. Reducing acute adverse outcomes in youths with type 1 diabetes: a randomized, controlled trial. Pediatrics. 2003;112(4):914–22. 14523186 10.1542/peds.112.4.914

[35] Holmes CS, Chen R, Mackey E, Grey M, Streisand R. Randomized clinical trial of clinic-integrated, low-intensity treatment to prevent deterioration of disease care in adolescents with type 1 diabetes. Diabetes Care. 2014;37(6):1535–43. 24623027 10.2337/dc13-1053 PMC4030089

[36] Katz ML, Volkening LK, Butler DA, Anderson BJ, Laffel LM. Family-based psychoeducation and care ambassador intervention to improve glycemic control in youth with type 1 diabetes: a randomized trial. Pediatr Diabetes. 2014;15(2):142–50. 23914987 10.1111/pedi.12065 PMC3915039

[37] Murphy HR, Wadham C, Hassler-Hurst J, Rayman G, Skinner TC. Randomized trial of a diabetes self-management education and family teamwork intervention in adolescents with Type 1 diabetes. Diabet Med. 2012;29(8):e249–54. 22507080 10.1111/j.1464-5491.2012.03683.x

[38] Butler AM, Hilliard ME, Christopher K, Baudino M, Minard C, Karaviti L. Feasibility and acceptability of the TEAM pilot trial with African American and Latino families. J Pediatr Psychol. 2025. 10.1093/jpepsy/jsaf001 PMC12225676 40139919

[39] Ellis DA, Frey MA, Naar-King S, Templin T, Cunningham P, Cakan N. Use of multisystemic therapy to improve regimen adherence among adolescents with type 1 diabetes in chronic poor metabolic control: a randomized controlled trial. Diabetes Care. 2005;28(7):1604–10. 15983308 10.2337/diacare.28.7.1604

[40] Ellis D, Naar-King S, Templin T, Frey M, Cunningham P, Sheidow A, et al. Multisystemic therapy for adolescents with poorly controlled type 1 diabetes: reduced diabetic ketoacidosis admissions and related costs over 24 months. Diabetes Care. 2008;31(9):1746–7. 18566340 10.2337/dc07-2094 PMC2518338

[41] Ellis DA, Templin T, Naar-King S, Frey MA, Cunningham PB, Podolski C-L, et al. Multisystemic therapy for adolescents with poorly controlled type I diabetes: Stability of treatment effects in a randomized controlled trial. J Consult Clin Psychol. 2007;75(1):168. 17295576 10.1037/0022-006X.75.1.168

[42] Wagner DV, Barry SA, Stoeckel M, Teplitsky L, Harris MA. NICH at its best for diabetes at its worst: Texting teens and their caregivers for better outcomes. J Diabetes Sci Technol. 2017;11(3):468–75. 28745094 10.1177/1932296817695337 PMC5505437

[43] Wagner DV, Barry S, Teplitsky L, Sheffield A, Stoeckel M, Ogden JD, et al. Texting adolescents in repeat DKA and their caregivers. J Diabetes Sci Technol. 2016;10(4):831–9. 27030051 10.1177/1932296816639610 PMC4928235

[44] Wong JC, Reed A, Noya C, Stone A, Spiro K, McGrath M, et al. Underresourced youth with diabetes in a community-based intervention show improved diabetes outcomes, technology use, and psychosocial functioning. Endocr Pract. 2025;31(5):571–7. 39956297 10.1016/j.eprac.2025.02.008

[45] Stover-Kempers SM, Torres KA, Barry-Menkhaus SA, Jenisch C, Spiro K, Harris MA et al. Health equity intervention for youth with type 1 diabetes and high social risk. Child (Basel). 2025;12(2). 10.3390/children12020200 PMC11854909 40003302

[46] Wagner DV, Mijanovic T, Barry S, Spiro K, Harris MA. Youth with Type 1 Diabetes and social risk: Intervention evaluation. Population Health Equity & Outcomes. In.

[47] Norman MZ, Torres KA, Saldana L, Spiro K, Naranjo D, Wong JC et al. Addressing disparities across systems: initial implementation of novel interventions in children’s healthcare (NICH). Global Implementation Research and Applications. 2025:1–15.

[48] Barry SA, Teplitsky L, Wagner DV, Shah A, Rogers BT, Harris MA. Partnering with insurers in caring for the most vulnerable youth with diabetes: NICH as an integrator. Curr Diab Rep. 2017;17(4):26. 28321766 10.1007/s11892-017-0849-4

[49] Ellis DA, Janisse H, Naar-King S, Kolmodin K, Jen K-LC, Cunningham P, et al. The effects of multisystemic therapy on family support for weight loss among obese African-American adolescents: findings from a randomized controlled trial. J Dev Behav Pediatr. 2010;31(6):461–8. 20585269 10.1097/DBP.0b013e3181e35337

[50] Wong JJ, Alamarie SA, Hanes SJ, Flores H, Ngo J, Schneider-Utaka AK et al. An RCT of a family intervention for adolescents living with type 1 diabetes: Who benefits most? Diabet Med. 2025:e70151. 10.1111/dme.70151 PMC12766931 41159277

[51] Hilliard ME, Cao VT, Eshtehardi SS, Minard CG, Saber R, Thompson D, et al. Type 1 doing well: Pilot feasibility and acceptability study of a strengths-based mHealth app for parents of adolescents with Type 1 diabetes. Diabetes Technol Ther. 2020;22(11):835–45. 32379496 10.1089/dia.2020.0048 PMC7698853

[52] Ellis D, Carcone AI, Templin T, Evans M, Weissberg-Benchell J, Buggs-Saxton C, et al. Moderating effect of depression on glycemic control in an ehealth intervention among black youth with type 1 diabetes: Findings from a multicenter randomized controlled trial. JMIR Diabetes. 2024;9:e55165. 38593428 10.2196/55165 PMC11040442

[53] Holtz B, Mitchell KM, Holmstrom AJ, Cotten SR, Dunneback JK, Jimenez-Vega J, et al. An mHealth-based intervention for adolescents with type 1 diabetes and their parents: Pilot feasibility and efficacy single-arm study. JMIR Mhealth Uhealth. 2021;9(9):e23916. 34519670 10.2196/23916 PMC8479605

[54] Patton SR, Monzon AD, Marker AM, Clements MA. A nonrandomized pilot of a group, video-based telehealth intervention to reduce diabetes distress in parents of youth with type 1 diabetes mellitus. Can J Diabetes. 2022;46(3):262–8. 35568427 10.1016/j.jcjd.2021.10.007 PMC9107594

[55] Flores Garcia J, Reid MW, Pyatak EA, Fox DS, Bisno DI, Salcedo-Rodriguez E, et al. Person-centered care and telehealth reduce distress and A1C for marginalized adolescents and young adults with type 1 diabetes. Clin Diabetes. 2025;43(2):270–81. 40303628 10.2337/cd24-0081 PMC12019005

[56] Bisno DI, Reid MW, Pyatak EA, Flores Garcia J, Salcedo-Rodriguez E, Torres Sanchez A, et al. Virtual peer groups reduce HbA1c and increase continuous glucose monitor use in adolescents and young adults with type 1 diabetes. Diabetes Technol Ther. 2023;25(9):589–601. 37335751 10.1089/dia.2023.0199

[57] Pyatak EA, Lee PJ, Nnoli ND, Mo Y, Khurana A, Ali A, et al. Telehealth occupational therapy improves psychosocial well-being but not glycemia among young adults with type 1 diabetes: The Resilient, Empowered, Active Living-Telehealth (REAL-T) randomized controlled trial. Diabetes Res Clin Pract. 2025;221:112005. 39884514 10.1016/j.diabres.2025.112005 PMC11991672

[58] Ellis DA, Idalski Carcone A, Ondersma SJ, Naar-King S, Dekelbab B, Moltz K. Brief computer-delivered intervention to increase parental monitoring in families of African American adolescents with type 1 diabetes: a randomized controlled trial. Telemedicine e-Health. 2017;23(6):493–502. 28061319 10.1089/tmj.2016.0182 PMC5488253

[59] Flores Garcia J, Reid MW, Torres Sanchez A, Ruelas V, Salvy SJ, Thomas A et al. Insights from team clinic: A person-centered virtual peer group care model adapted for marginalized and historically excluded youth with type 1 diabetes (T1D). Child (Basel). 2024;11(11). 10.3390/children11111383 PMC11592975 39594958

[60] Amirav I, Vandall-Walker V, Rasiah J, Saunders L. Patient and researcher engagement in health research: a parent’s perspective. Pediatrics. 2017;140(3):e20164127. 28851740 10.1542/peds.2016-4127

[61] Goodman MS, Sanders Thompson VL. The science of stakeholder engagement in research: classification, implementation, and evaluation. Translational Behav Med. 2017;7(3):486–91. 10.1007/s13142-017-0495-z PMC5645283 28397159

[62] Ramanadhan S, Alemán R, Bradley CD, Cruz JL, Safaeinili N, Simonds V, Aveling EL. Using participatory implementation science to advance health equity. Annu Rev Public Health. 2024;45. 10.1146/annurev-publhealth-060722-024251 PMC11251496 38109515

[63] Nápoles AM, Stewart AL. Transcreation: an implementation science framework for community-engaged behavioral interventions to reduce health disparities. BMC Health Serv Res. 2018;18(1):710. 30208873 10.1186/s12913-018-3521-z PMC6134771

[64] Garcia JF, Reid MW, Raymond JK. Designing, implementing, and adapting virtual care models for marginalized communities. Endocrinol Metabolism Clin. 2025;54(2):315–28. 10.1016/j.ecl.2025.02.018 40348572

[65] Salvy SJ, Ruelas V, Majidi S, Thomas A, Ashwal G, Reid M, et al. Team clinic: Expansion of a multidisciplinary care model for adolescents with type 1 diabetes. Contemp Clin Trials. 2020;95:106079. 32634486 10.1016/j.cct.2020.106079 PMC8098333

[66] Butler AM, Hilliard ME, Comer-HaGans D. Review of community-engaged research in pediatric diabetes. Curr Diab Rep. 2018;18(8):56. 29931496 10.1007/s11892-018-1029-x PMC6512775

[67] Ellison VN, Desai KR, Eddington AR, Berlin KS. Commentary: Breaking barriers, centering community voices, and advancing equitable diabetes care for Black and Latine families—lessons from the TEAM intervention. J Pediatr Psychol. 2025;50(5):399–401. 40373329 10.1093/jpepsy/jsaf040

[68] Akturk HK, Agarwal S, Hoffecker L, Shah VN. Inequity in racial-ethnic representation in randomized controlled trials of diabetes technologies in type 1 diabetes: Critical need for new standards. diabetes care. 2021;44(6):e121–3. 34016613 10.2337/dc20-3063 PMC8247501

[69] Price J, Yang C, Thomas C, Deatrick J, Butler A, Rogers M, Rogers I, Rogers M, Byrne A, Byrne O, Cammarata C, Shoe K, Deemedio M, Gannon A, Kazak AE. (June 2025). Strategies to increase inclusion in pediatric type 1 diabetes psychosocial intervention studies. Poster presentation at the American Diabetes Association 85th Scientific Sessions, Chicago, IL.

[70] Butler AM, Hilliard ME, Fegan-Bohm K, Minard C, Anderson BJ. Peer-support intervention for African American and Latino parents to improve the glycemic control trajectory among school-aged children with type 1 diabetes: A pilot and feasibility protocol. Contemp Clin Trials. 2022;116:106739. 35341991 10.1016/j.cct.2022.106739 PMC9295821

[71] Vander Wyst KB, Olson ML, Hooker E, Soltero EG, Konopken YP, Keller CS, Shaibi GQ. Yields and costs of recruitment methods with participant phenotypic characteristics for a diabetes prevention research study in an underrepresented pediatric population. Trials. 2020;21(1):716. 32799920 10.1186/s13063-020-04658-8 PMC7429699

[72] Medina Peñaranda R, Figg LE, Hanes SJ, Shaw GM, Chamberlain LJ, Raymond J, Addala A. Strategies for equitable recruitment to engage underrepresented youth and their families into clinical research: findings from the BEAD-T1D pilot study. Hormone Res Paediatrics. 2026;99(1):122–30. 10.1159/000541774 PMC11968438 39369697

